# Effect of pH shift and high pressure homogenization on the structure and techno-functional properties of protein extracts from substandard peas

**DOI:** 10.1016/j.crfs.2025.101286

**Published:** 2025-12-20

**Authors:** Stella Plazzotta, Sofia Melchior, Lorenzo Barozzi, Alberto Saitta, Maria Cristina Nicoli, Lara Manzocco

**Affiliations:** a Department of Agricultural, Food, Environmental and Animal Sciences, University of Udine, Via Sondrio 2/A, Udine, 33100, Italy; b Department of Human Sciences and Promotion of the Quality of Life, San Raffaele University, Rome, Italy

**Keywords:** Upcycling, Plant proteins, Protein structure, Functional properties, Solubility

## Abstract

This study aimed to elucidate the mechanisms involved in the modification of structure and techno-functional properties of pea proteins upon pH-shift (PS), high-pressure homogenization (HPH) and their combination (PS + HPH). Unlike many previous studies on dried commercial isolates, treatments were applied directly to wet extracts to increase sustainability. Wet protein extracts obtained from substandard peas by alkaline extraction and isoelectric precipitation were subjected to PS, HPH and PS + HPH before drying. Differential scanning calorimetry showed that proteins were extensively denatured already upon extraction. None of the treatments impacted protein primary and secondary structure, as detected by SDS-PAGE and FTIR. Single treatments promoted protein aggregate detachment, exposing hydrophilic and hydrophobic groups, and particle size decrease, while PS + HPH favored intramolecular bonding of the small particles formed upon PS. These structural changes increased extract solubility, interfacial properties, water/oil holding capacity and antioxidant activity. PCA highlighted structural changes induced by PS and HPH on pea proteins to be associated with improved functionalities, opening new possibilities for pea waste upcycling into protein-rich ingredients.

## Highlights

• Proteins were extracted from substandard peas wasted by canning industry.
• pH shift-PS and high pressure homogenization-HPH were applied to wet protein extracts.
• Extraction caused extensive denaturation and aggregation of pea proteins.
• PS and HPH application resulted in protein aggregate detachment.
• PS and HPH lead to proteins with improved techno-functionalities from wasted peas.

## Graphical abstract

**Figure:**
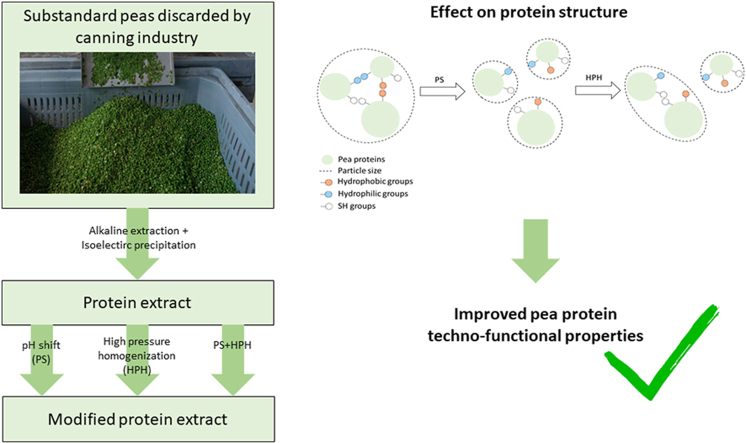

## 1 Introduction

The world's population is estimated to reach 9.7 billion people in 2050, with a 56 % upsurge in demand for food, and more specifically for proteins (van Dijk et al., 2021). Animal protein production has almost reached its maximum capacity (Boland et al., 2013) and cannot satisfy such a request alone (Foegeding, 2015; Loveday, 2019; Pojić et al., 2018). Furthermore, the production chains of animal proteins have a significant environmental impact due to the high consumption of land, feed, and water associated with high emissions of greenhouse gases (Aschemann-Witzel et al., 2021).

In this context, recent years have witnessed a so-defined “protein transition”, aiming to rebalance protein production between animal and plant proteins and gaining momentum in scientific and policy discussions on food system transformation (Aiking and de Boer, 2020; Gonera et al., 2021).

To meet the consequent increased demand for plant protein-rich foods, legume production has been constantly increasing in the last years (Prudhomme et al., 2020). Among all, pea has been emerging as one of the most important crops grown worldwide and in Europe (Rogers et al., 2024), thanks to the high protein content (18–30 g/100 g dry matter) and low allergenicity (Taylor et al., 2021). Yellow peas (*Pisum sativum L.*) are marketed in dried form (dried peas), while green peas (*Pisum sativum L. var. macrocarpon*) are marketed as fresh, frozen or canned (Tassoni et al., 2020). Green pea processing is particularly challenging for industries since it is concentrated between 12 and 15 weeks after harvesting, during which large amounts of fresh peas need to be transformed. During the pea processing campaign, huge quantities of waste are generated at the industry level. This waste is mostly represented by the so-defined “substandard peas”, i.e., seeds not meeting the quality standards of color, shape, and size for further processing. Based on the estimation of a large Italian canning company, substandard seeds represent up to 15 % of overall processed peas, accounting for 0.135 million tons/year waste in EU only. However, substandard peas present the same composition as standard ones, thus representing a low-cost source of plant proteins potentially able to support the plant protein transition (Manzocco et al., 2024).

As is well known, plant proteins often present reduced functional properties compared to their animal counterpart (McClements and Grossmann, 2021), being characterized, for instance, by poor solubility, interfacial properties, and water and oil holding capacity (Day et al., 2022). Several technological strategies have thus been proposed to modify the structure of plant proteins and improve their functionalities (Nikbakht Nasrabadi et al., 2021). In this context, pH-shift (PS) and high-pressure homogenization (HPH) treatments have shown promising results (D'Alessio et al., 2023; Ghorbani et al., 2025; Guo et al., 2021; Jiang et al., 2018; Ma et al., 2023; Ong et al., 2022; Saricaoglu, 2020; Yildiz, 2018; Yildiz & Yildiz, 2023; Yildiz, 2025). PS exploits the exposure of plant proteins to pH conditions far below (or above) their isoelectric point (pI), to make them highly positively (or negatively) charged, followed by neutralization (Jiang et al., 2018). This induces protein quaternary structure disruption and the release of individual monomers (Tang, 2021). Moreover, at extreme pH values, proteins partially unfold so that, when the pH is restored to neutrality, re-folding occurs leading to secondary and tertiary structures other than those of the native protein (Jiang et al., 2018). For instance, when applied to protein isolates from pea, quinoa, soy, sesame and pumpkin, PS treatments were demonstrated to induce the exposure of side chains originally buried in the protein core, leading to an increase in both water solubility and interfacial properties (Ghorbani et al., 2025; Jiang et al., 2018; Yildiz, 2018; Yildiz & Yildiz, 2023).

HPH is based on the application of hydrodynamic pressures up to 400 MPa to a fluid sample that is forced to pass through a micrometric homogenization valve. During this process, the fluid undergoes significant mechanical, elongational and shear stresses, as well as cavitation (Paquin, 1999), which lead to the modification of protein structure and thus functionalities. The mechanical effects produced by HPH have been reported to significantly reduce the dimension of protein particles, as well as unfold and alter the secondary, tertiary, and quaternary structures that control protein inter/intra-molecular interactions and surface activity (Saricaoglu, 2020; Tabilo-Munizaga et al., 2019). HPH has been reported to increase lentil, pea, and soy protein isolate solubility (D'Alessio et al., 2023; Luo et al., 2022b; Saricaoglu, 2020), as well as oil holding capacity (OHC) and emulsifying properties of pea, chickpea, bean and quinoa protein isolates (Guo et al., 2021; Luo et al., 2022a; Ma et al., 2023; Ong et al., 2022).

PS and HPH have also been combined in some recent studies, yielding notable effects on protein structure and functionality. In particular, the combination of these treatments led to a higher improvement in the functional properties of proteins isolated from soy, quinoa, hempseed, melon seeds and lentils, compared to the application of single PS or HPH treatments (Parlak et al., 2024; Yildiz et al., 2017; Yildiz, 2025; Yildiz & Yildiz, 2023).

Most of the studies addressing the effect of PS and HPH on protein structure and technological properties have been conducted using commercial protein isolates. The latter are produced through multi-step processes involving large amounts of water and are finally dried to obtain a powder. The powder is then functionalized by PS and/or HPH upon rehydration, adding further water. Nevertheless, to increase the sustainability of real industrial processes and avoid inefficient water cycles, PS and HPH treatments should be applied to raw/fresh vegetable materials, rather than to dried protein extracts, especially starting from low-cost raw materials, such as substandard peas. Moreover, commercial protein extracts used in the literature are obtained by the application of rather different production processes (Barozzi et al., 2025; Boye et al., 2010; Cui et al., 2020; Lam et al., 2018), and subjected to spray-drying, during which protein denaturation is likely to occur. As a result, commercial products with considerably different techno-functional properties are obtained (Corredig et al., 2020; D'Alessio et al., 2023; Geerts et al., 2017; Stone et al., 2015), making the effect of PS and HPH treatments on protein structure and functionality difficult to be unambiguously assessed.

That said, the open question remains whether HPH and PS post-extraction treatments can further modify the techno-functional properties of already extensively denatured proteins. Based on the literature, it could be inferred that PS may promote the disruption of weak electrostatic interactions within aggregates of denatured proteins, whereas HPH may primarily induce their mechanical fragmentation (Saricaoglu, 2020; Tabilo-Munizaga et al., 2019).

Given these considerations, this study aimed to evaluate the effect of PS and HPH, applied individually or in combination, on the structural and techno-functional properties of protein extracts obtained from substandard peas. To this aim, proteins were extracted from industry-generated substandard peas. The wet extract was treated using PS, HPH, or their combination (PS + HPH), and subjected to freeze-drying. The dried extracts were then analyzed for gross composition, structural properties (SDS-PAGE, FTIR, DSC, particle size distribution, soluble protein content, free SH group, surface hydrophobicity) and techno-functional properties (solubility, interfacial properties, and water and oil holding capacity).

## 2 Materials and methods

### 2.1 Materials

Substandard peas were kindly supplied by Conserve Italia Soc. Coop. Agricola (Pomposa, Italy). The reagents used for this study were: sodium dodecyl sulphate (SDS), sodium phosphate dihydrate, tris-base, 8-anilinonaphthalen-1-sulfonic acid (ANS), phosphate buffer saline (PBS), sulfuric acid, bicinchoninic acid (BCA), Tris Base, dietary fibre assay kit, methanol, cupric sulphate, acetic acid (Sigma-Aldrich, St. Louis, Missouri, USA); 2,2′-dinitre-5,5′-iodinedbenzoic acid (DNTB) (Acros, New Jersey, USA); dimethylformamide (Jassen, Geel, Belgium); potassium dihydrogen phosphate, potassium hydrogen phosphate, copper sulphate (J.T. Baker, Deventer, Netherlands); sodium dihydrogenophosphate (Prolabo 54, Fontenay, France); sodium hydrogen phosphate, potassium, sulphate, glycine, boric acid, diethyldiaminootetraacetic acid (EDTA), hydrochloric acid (HCl), glycine, sodium hydroxide (NaOH) (Carlo Erba, Milan, Italy). Laemmli sample buffer 2 × , β-mercaptoethanol, Coomassie blue, Mini-PROTEAN TGX Stain-Free Gels (Bio-Rad Laboratories, Inc., Hercules, CA, USA); 2,2-diphenyl-1-picrylhydrazyl (DPPH) (Tokyo Chemical Industry, Zwijndrecht, Belgium); Trolox (Cayman Chemical Company, Michigan, USA). Sunflower oil was purchased in a local market. Milli Q water (System advantage A10, Millipore S.A.S., Moldheim, France) was used for all the experiments.

### 2.2 Protein extraction from substandard peas

The substandard fresh peas were manually selected to remove pods, leaves and insects, blanched in water at 90 °C for 3 min, quickly frozen for 30 min in a blast chiller (AOFPS061, Electrolux, Pordenone, Italy) set to −42 °C and stored at −20 °C (REX71FF, Electrolux, Pordenone, Italy) until use. After thawing overnight at 4 °C, the peas were ground in water (20 g/100 g) with a domestic mixer for 60 s (Bosch MS6CM6120 Minipimer 1000W, Gerlingen, Germany), followed by further grinding for 4 min at 10,000 rpm (T 18 digital ULTRA-TURRAX®, IKA, Milan, Italy).

The suspension was then subjected to protein extraction by alkaline extraction followed by isoelectric precipitation using a modified method of Boye et al. (2010). To this aim, the pH of the suspension was raised to 12.0 ± 0.1 by the addition of NaOH 6 M, stirred at 50 °C for 1 h on a magnetic stirrer (IKA C-MAG HS7, Staufen im Breisgau, Germany) and then centrifuged (Next J-25, Beckman, Fullerton, California) at 10,000×*g* at 4 °C for 30 min. The supernatant containing the proteins was recovered and its pH was adjusted to 4.5 ± 0.1 with HCl 6 M. The suspension was left to rest for 1 h at room temperature and then centrifuged at 10,000×*g* at 4 °C for 30 min, to recover the precipitated proteins.

### 2.3 Treatments on wet pea protein extracts

The wet protein extracts were diluted 1:5 (weight basis) with distilled water and then subjected to the different procedures described in the following.

For the control sample, the diluted extract was neutralized to pH 7.0 ± 0.1 with NaOH 1 M.

The diluted extract was subjected to PS treatment as previously reported (Yildiz and Yıldız, 2023), with minor modifications. To this aim, the pH was adjusted at 12.0 ± 0.1 by adding 6 or 1 M NaOH, maintained under agitation at 50 °C for 1 h, and finally neutralized at pH 7.0 ± 0.1.

For HPH treatment the method of Melchior et al. (2022) was adapted; the diluted extract was cooled (10 ± 1 °C) in an ice bath to prevent temperature rise during the treatment (TFA Dostmann GmbH & Co. KG, Wertheim, Germany). A high-pressure homogenizer (Panda Plus 2,000, GEA Niro Soavi Spa, Parma, Italy) equipped with two tungsten carbide valves and a flow rate of 10 L/h was used. The first valve was set to a pressure of 150 MPa, while the second one was set to a constant pressure of 5 MPa.

To understand the effect of the combined treatment, the diluted extract was subjected to PS treatment, cooled at 10 ± 1 °C in an ice bath and subjected to the HPH treatment (PS + HPH).

The treated samples were finally freeze-dried (Mini-Fast Edwards, Milan, Italy). All the obtained freeze-dried pea protein extracts were packed in plastic bags under vacuum, without modifying the atmosphere within the pack, and stored in desiccators until use.

### 2.4 Characterization of pea protein extracts

#### 2.4.1 Protein content

Protein content was evaluated through nitrogen Kjeldahl determination (N × 5.40) (Sosulski and Imafidon, 1990).

#### 2.4.2 Dietary fiber content

Total dietary fibre (TDF), soluble dietary fibre (SDF), and insoluble dietary fibre (IDF) contents were measured by using the total dietary fibre assay kit and following the enzymatic-gravimetric official method 985.29–1986 (AOAC, 2003).

#### 2.4.3 Sodium dodecyl sulphate - PolyAcrylamide gel electrophoresis (SDS-PAGE)

SDS-PAGE was performed according to the method of Laemmli (1970). Ten mg of freeze-dried extract was mixed with 500 μL Laemmli sample buffer 2 × containing 2.0 mL/100 mL of β-mercaptoethanol. The mixtures were incubated at room temperature for 1 h, then heated at 90 °C for 5 min in a water bath and centrifuged (Mikro 120, Hettich Italia srl, Milan, Italy) at 9,500×*g* for 10 min at 20 °C. The running buffer was prepared mixing 14.4 g/100 mL glycine, 3 g/100 mL Tris Base, and 1 g/100 mL SDS in deionized water. Ten μL of sample was loaded onto polyacrylamide gels. The run was performed on a Mini-PROTEAN® Tetra Cell apparatus (Bio-Rad, Hercules, CA, USA) at a constant amperage of 30 mA. Subsequently, the gel was placed for 30 min in a gel-fixing aqueous solution, containing 40 mL/100 mL methanol and 10 mL/100 mL acetic acid, and stained with Coomassie blue overnight. The gels were destained with water for 30 min, and images were acquired with a gel documentation system G:BOX (Chemi XX9, Syngene, Cambridge, UK). A molecular weight (MW) standard consisting of a cocktail of ten proteins with known MWs, range 10–250 kDa, was used to indicate the MW protein of the sample.

#### 2.4.4 Fourier transform infrared spectroscopy (FTIR) measurement

FTIR spectra were recorded at 25 ± 1 °C by using a FTIR instrument, equipped with an ATR accessory and a Zn-Se crystal that allows collection of FTIR spectra directly on freeze-dried sample without any special preparation (Alpha-P, Bruker Optics, Milan, Italy). The “pressure arm” of the instrument was used to apply constant pressure to the sample, positioned onto the Zn–Se crystal, to ensure good contact between the sample and the incident IR beam. All FTIR spectra were collected in the range from 4000–400 cm^−1^, at a spectrum resolution of 4 cm^−1^ and with 32 co-added scans. A background scan of the clean Zn–Se crystal was acquired prior to sample scanning. The collected FTIR spectra were pre-processed (baseline corrected, smoothened, and normalised) using the OPUS software (version 7.0 for Microsoft Windows, Bruker Optics, Milan, Italy) and Gaussian curve fitting of deconvoluted amide I (1600–1700 cm^−1^) was performed using Origin Pro 9 (OriginLab, Northampton, MA, USA) accordingly with Sow and Yang (2015). The fitting quality of the Gaussian curves was confirmed by having R^2^ > 0.997. The attribution of the peak to the secondary structure was performed according to Kong and Yu (2007).

#### 2.4.5 Differential scanning calorimetry (DSC)

DSC analysis was carried out using the DSC 3 Star^e^ System differential scanning calorimeter (Mettler-Toledo, Greifensee, Swiss). Heat flow calibration was achieved using indium (heat of fusion 28.45 J/g). Temperature calibration was carried out using hexane, water and indium (having melting points of −93.5 °C, 0.0 °C and 156.6 °C, respectively). Freeze-dried protein extracts were dispersed at 10 g/100 mL in PBS (50 mM; pH 7.2 ± 0.1) and stirred overnight. Then, approximately 20 mg of the dispersion were loaded in 100 μL aluminum DSC pans, closed with hermetic sealing, heated from 10 °C to 105 °C at 10 °C/min under nitrogen flow (20 mL/min), and using an empty pan as a reference (D'Alessio et al., 2023). The denaturation temperature and associated enthalpy were obtained by using the program STAR^e^ ver. 16.10 (Mettler-Toledo, Greifensee, Switzerland).

#### 2.4.6 Soluble protein content

The soluble protein content of the freeze-dried pea protein extracts was quantified by the BCA assay according to Smith et al. (1985), adapted to a 96-well microplate spectrophotometer procedure. The BCA working reagent (WR) was prepared by mixing the bicinchoninic acid solution with the cupric sulphate solution (4 g/100 mL) to reach the final ratio of 50:1. Freeze-dried pea protein extracts were solubilized in water (0.5 g/100 g), stirred overnight at room temperature, and centrifugated at 20,000×*g* for 20 min at 4 °C to separate the supernatant. Aliquots of 25 μL of supernatant were placed into a 96-well MicrotiterTM microplates (Thermo Fisher Scientific, Waltham, MA USA) and 200 μL of WR was added to each well. Samples were incubated in a microplate reader (Sunrise-Basic Tecan, Tecan GmbH, Grödig, Austria) at 37 °C for 30 min in the dark. Then the absorbance was measured at 562 nm and soluble protein content was determined by comparison with the calibration curve prepared by bovine serum albumin (BSA) (0–2000 μg/mL, R^2^ = 0.994).

#### 2.4.7 Free SH groups

The free SH groups of 5 mg freeze-dried pea protein extracts were determined according to Ellman's assay (Ellman, 1959), following the methodology described by Panozzo et al. (2014). Free SH groups were expressed as μmol/g_extract_.

#### 2.4.8 Surface hydrophobicity

Surface hydrophobicity was determined by fluorescent testing according to the method of Cui et al. (2012). A protein dispersions at 2 g/100 mL in 50 mM phosphate buffer was prepared and diluted 1:2, 1:5, 1:10, 1:20 (v/v). After the complete hydration of the sample by overnight stirring at 4 °C, 0.6 mL of the fluorescent probe ANS (8 mM in 50 mM phosphate buffer, pH 6.8 ± 0.1) was added to 10 mL of samples and let react for 15 min in a dark place. A reference blank was also prepared without the sample. The fluorescence spectrometer (Cary Eclipse model, Agilent Technologies, Santa Clara, CA, United States) used to determine the relative fluorescent intensity (RFI) of each sample was set as follows: excitation at 390 nm and emission at 480 nm. RFI is defined by the following equation (Eq. (1)):

(Eq. 1) $$RFI=\frac{\left(F-F_{0}\right)}{F_{0}}$$

where F is the fluorescence intensity of samples and $F_{0}$ is the fluorescence intensity of the reference blank. The surface hydrophobicity index (H_0_) is determined from the slope obtained by plotting RFI versus protein concentration.

#### 2.4.9 Particle size

The particle size distribution was measured by dynamic laser light scattering (DLS, Zetasizer NanoZS, Malvern Instruments, Worcestershire, UK). The freeze-dried pea protein extracts were diluted (1 g/100 mL) with deionized water and placed in a cuvette where the laser light, set at 173° angle, was scattered by the particles. Particle size was reported as volume-weighed mean diameter in nm.

### 2.5 Techno-functionalproperties

#### 2.5.1 Total solubility

Freeze-dried pea protein extracts ($M_{1}$) were suspended in deionized water (0.5 g/100 g), stirred at room temperature overnight and then centrifuged (Mikro 120, Hettich Italia srl, Milan, Italy) at 20,000×*g* for 20 min at 4 °C (Plazzotta et al., 2021). The supernatant was eliminated, and the insoluble precipitate was dried in a vacuum oven (Vuotomatic 50, Bicasa, Milan, Italy) overnight and exactly weighted ($M_{2}$). Sample solubility was calculated by the following equation (Eq. (2)).

(Eq. 2) $$Total\;solubility\;\left(\%\right)=\frac{M_{1}-M_{2}}{M_{1}}\;\cdot \;100$$

#### 2.5.2 Emulsifying properties

Freeze-dried pea protein extracts were dispersed in deionized water (1 g/100 mL) and stirred overnight at 4 °C. The prepared water phase was then added with sunflower oil (9:1 w/v water phase:oil ratio) and homogenised with an Ultraturrax (T 18 digital ULTRA-TURRAX®, IKA, Milan, Italy) at 1,000×*g* for 3 min. A volume of 250 μL of the obtained emulsions was taken from the bottom of the container, diluted (1 mL/100 mL) in SDS solution (0.1 g/100 mL), and used to determine emulsifying activity (EAI) and stability (ESI) indices at 0 and 60 min respectively by spectrophotometric analysis at 500 nm. Indices were calculated as follows (Eqs. (3) and (4)):

(Eq. 3) $$EAI\left(\frac{m^{2}}{g}\right)=\frac{2\times 2.303\times A\times DF}{C\times \left(1-\theta \right)\times \Phi \times 10,000}$$

(Eq. 4) $$ESI\left(\min\right)=\frac{A_{0}}{A_{0}-A_{60}}\cdot 60$$

where DF is the dilution factor (100), C is the initial protein concentration (0.01 g/mL), θ is the emulsion oil fraction (0.1), Φ is the optical path (1 cm), $A_{0}$ and $A_{60}$ are the absorbances of the diluted emulsion at time 0 and after 60 min, respectively.

#### 2.5.3 Foaming properties

Freeze-dried pea protein extracts were suspended in distilled water (1 g/100 mL) and stirred at room temperature for 60 min. Aliquots of 10 mL suspension (V_i_) were foamed (T 18 digital ULTRA-TURRAX®, IKA, Milan, Italy) for 3 min at 800×*g* in a graduated cylinder and the volume of foam was measured just after preparation (V_0_) and after 30 min (V_30_). The foaming activity index (FAI) and the foaming stability index (FSI) were computed using the following equations (Eqs. (5) and (6)).

(Eq. 5) $$FAI\;\left(\%\right)=\frac{V_{0}-V_{i}}{V_{0}}\;\cdot \;100$$

(Eq. 6) $$FSI\;\left(\%\right)=\frac{V_{30}}{V_{0}}\;\cdot \;100$$

#### 2.5.4 Water and oil holding capacity (WHC and OHC)

WHC and OHC were evaluated according to the method of Sen and Kahveci (2020) with minor modifications. Freeze-dried pea protein extracts were suspended in distilled water or sunflower oil (0.1 g/mL), stirred using a vortex (Vortex 1, Ika, Milan, Italy) for 30 s and centrifuged for 20 min at 20,000×*g* at 4 °C. The supernatant was eliminated, and the pellet was weighted. Water and oil holding capacity (WHC and OHC) were calculated as the amount of water or oil held by 1 g of sample (Manzocco et al., 2024).

#### 2.5.5 Antioxidant activity (AA)

The 1,1- diphenyl-2-picrylhydrazil assay (DPPH) was used, according to the method previously described (Paciulli et al., 2023) with some modifications. The freeze-dried protein extracts were suspended in water and stirred (600 rpm for 120 min) at room temperature. A methanolic solution of DPPH 1 mM was prepared in a flask, stirred for 2 h in dark and diluted (1 mL/10 mL) with methanol before the analysis. For the analysis, 1500 μL of DPPH and 500 μL of the protein extract aqueous dispersion were added to a 4 mL cuvette, which was covered with parafilm and incubated in the dark at room temperature for 30 min. Then, sample absorption at 517 nm was measured using a spectrophotometer UV–Vis (Thermo Scientific Waltham, Massachusetts, United States). To prepare the blank, extract water suspension was replaced with 50 μL of a methanol-distilled water 70:30 (volume basis) solution. The antioxidant activity (AA) of the samples was quantified based on the comparison with a calibration curve prepared using a stock solution of Trolox 25 μM dissolved in a 70 mL/100 mL methanolic solution, from which dilutions from 20 μM to 1 μM were prepared. AA was expressed as μg_TE_/g of protein extract, where TE stays for Trolox equivalents.

### 2.6 Statistical analysis

All determinations were expressed as the mean ± standard deviation (SD) of at least three repeated measurements from two experiment replicates. Statistical analysis was performed by using R v. 4.1.3 (The R Foundation for Statistical Computing, Wien, Austria). Bartlett's test was used to check the homogeneity of variance, one-way ANOVA was carried out and Tukey test was used to determine statistically significant differences among means (p < 0.05). The principal component analysis (PCA) was conducted by Origin Pro 9 software (OriginLab, Northampton, MA).

## 3 Results and discussion

### 3.1 Effect of PS and HPH on pea protein extract composition and structure

Technological interventions of pH-shift (PS), high-pressure homogenization (HPH) and their combination (PS + HPH) were applied to the wet extracts obtained from substandard peas by alkaline extraction and isoelectric precipitation. The derived freeze-dried protein extracts showed a fiber and protein content of around 10 and 70 g/100 g_extract_, respectively (Supplementary Table S1). Literature reports that alkaline extraction can yield protein purities ranging from about 60 to over 90 g/100 g_extract_ (Das et al., 2021; Lqari et al., 2002; Peyrano et al., 2016; Ruiz et al., 2016), depending on several processing variables, including the physical state of the vegetable material (e.g., fresh vs dried) and particle size of the extracted material, the temperature and duration of the treatment, and the intensity of centrifugation. The protein purity (70 g/100 g_extract_) obtained in this study can be attributed to the fact that the applied extraction pH (pH 12) is known to solubilize not only proteins but also other cellular constituents such as carbohydrates and fibers (Liu et al., 2023; Silva et al., 2023).

#### 3.1.1 Primary structure

**Fig. 1:**
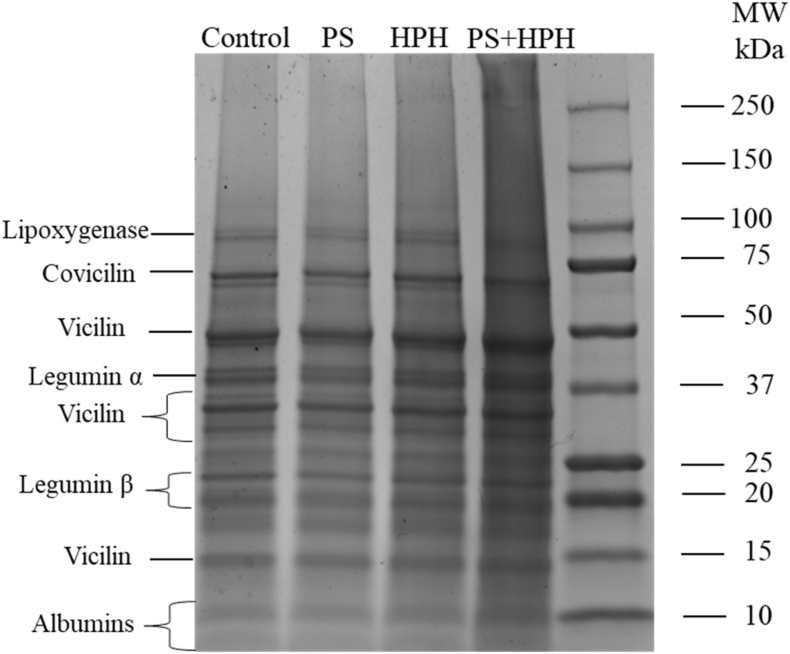
SDS-PAGE profiles of pea protein extracts subjected to pH shift (PS), high pressure homogenization (HPH), and their combination (PS + HPH). Data relevant to the untreated pea protein extract (control) are also shown.

SDS-PAGE was conducted to assess the effect of the technological interventions of PS, HPH and PS + HPH on pea protein primary structure (Fig. 1).

The SDS-PAGE detected the major storage pea proteins, including covicilin, with a molecular weight (MW) of about 75 kDa, vicilin, for which the 3 typical subunits were identified at about 50, 35 and 15 kDa, legumin subunits α and β with a MW of about 40 and 20, respectively, and albumins (MW 10 kDa), along with lipoxygenase (MW around 100 kDa) (Liang and Tang, 2013). The patterns did not significantly differ based on the applied treatment, confirming that the protein primary structure was not affected by PS, HPH or their combination. Similar results were obtained by Jiang et al. (2017) by the application of alkaline PS at pH 12 on a commercial pea protein isolate; accordingly, Parlak et al. (2024) showed that HPH up to 150 MPa did not influence the primary structure of proteins extracted from lentils.

#### 3.1.2 Secondary structure

To assess if the technological treatments affected pea protein secondary structure, Fourier-transform infrared spectroscopy (FTIR) was conducted, focusing on the wavelength region between 1600 and 1700 cm^−1^, corresponding to the Amide I band, related to the stretching vibrations of the C=O bond of the amide groups (Kong and Yu, 2007). Table 1 reports the percentage distribution of each secondary structure in the different samples.

**Table 1:** Relative content of secondary structures of pea protein extracts subjected to pH shift (PS), high pressure homogenization (HPH), and their combination (PS + HPH). Data relevant to the untreated pea protein extract (control) are also shown.

Treatment	α-helix	β-sheet	β-turn	Random coil
Control	22.6 ± 2.7 ^b^	62.3 ± 4.5^a^	4.8 ± 1.4 ^b^	10.3 ± 6.9^a^
PS	25.6 ± 3.0 ^b^	64.0 ± 1.2^a^	4.9 ± 0.9^a^	5.5 ± 1.8^a^
HPH	29.8 ± 3.0^a^	52.8 ± 3.5 ^b^	8.8 ± 3.0^a^	8.6 ± 2.7^a^
PS + HPH	25.6 ± 6.5^ab^	57.8 ± 7.2^ab^	7.3 ± 3.1^a^	9.3 ± 2.3^a^

^a-b^: in each column, different letters indicate significantly different mean values (p < 0.05).

It must be noted that a high degree of variability in the content of secondary structure in plant proteins is reported in the literature, due to differences in cultivar and/or extraction and/or purification and/or drying methods (Shevkani et al., 2019). Despite this, a higher proportion of β-conformations compared to α-conformations is reported for pea proteins (Shevkani et al., 2015), as also confirmed by data reported in Table 1, which show a predominance of β-sheets over α-helixes in the control pea protein extract. Overall, the effect of the applied treatments on pea protein secondary structure was negligible, in contrast with recent works relevant to PS- and HPH-induced modification of plant proteins extracted using alkaline extraction and isoelectric precipitation. For instance, PS and HPH have been reported to significantly impact the secondary structure of proteins extracted from hemp, rapeseed and pea (D'Alessio et al., 2023; Fan et al., 2020; Wang et al., 2024; Zhang et al., 2022; Zhi et al., 2022), respectively. The limited intramolecular changes at the secondary-structure level of pea proteins here considered may be due to the fact that the applied extraction procedure caused protein extensive denaturation.

#### 3.1.3 Thermal properties

To confirm the extensive denaturation undergone by pea proteins, DSC analyses were conducted.The DSC patterns of the control extract (Supplementary Fig. S1) showed a denaturation peak at 91.5 ± 0.9 °C, in agreement with the results of D'Alessio et al. (2023) and Kuang et al. (2023).

Nevertheless, the peak intensity was extremely low (48.6 ± 1.1 × 10^−3^ J/g), indicating that the proteins were extensively denatured, which is probably the outcome of alkaline extraction at high pH value (pH 12) and isoelectric precipitation applied in this study (Tanger et al., 2020). In this regard, the application of alkaline extraction at milder pH values (pH 8) has been reported to highly preserve the native structure of pea proteins (D'Alessio et al., 2023), leading to denaturation peak enthalpies higher than 10 J/g (Kuang et al., 2023). The application of PS and HPH, both as single interventions and in combination, resulted in flat DSC thermograms, suggesting that these pre-treatments further contributed to protein denaturation.

#### 3.1.4 Surface properties

Denaturation most commonly results in the exposure of specific residues on the protein surface, which can be investigated by assessing protein solubility, free sulfhydryl groups and surface hydrophobicity (Table 2).

**Table 2:** Soluble protein content, free SH groups and surface hydrophobicity (H_0_) of pea protein extracts subjected to pH shift (PS), high pressure homogenization (HPH), and their combination (PS + HPH). Data relevant to the untreated pea protein extract (control) are also shown.

Treatment	Soluble protein content (g/100 g_proteins_)	Free SH groups (μmol/g_extract_)	H_0_
Control	44.0 ± 2.3^c^	13.17 ± 0.76^c^	5184 ± 63^c^
PS	77.5 ± 3.7^a^	17.38 ± 0.74^a^	13547 ± 879^a^
HPH	64.0 ± 1.9^b^	15.21 ± 0.47^b^	7399 ± 16^b^
PS + HPH	71.8 ± 1.7^a^	14.16 ± 0.48^bc^	15036 ± 528^a^

^a-d^ means within the same column indicated by different letters are significantly different (p < 0.05).

The control extract presented a protein solubility lower than 45 %, probably associated with the extensive denaturation induced by the extraction process, as confirmed by the FTIR (Table 1) and DSC results (Paragraph 3.1.3). All the treatments significantly (p < 0.05) increased the extract content in soluble proteins, which indicates a major exposure of hydrophilic residues, such as -OH and charged groups, promoting protein-water interactions (Wouters et al., 2016). In particular, in the PS- and HPH-treated extracts, the soluble protein amount increased by 76 and 45 %, respectively, and intermediate results were obtained for the extract subjected to the combined treatment.

The application of PS resulted in the most significant increase in the concentration of free SH groups and hydrophobic residues, as indicated by the increased H_0_ value. A similar but less pronounced effect was also detected for HPH single treatment. These results are in line with those obtained by Luo et al. (2022a) and Jiang et al. (2018) on a quinoa protein extract subjected to HPH (10–50 MPa) and a pea protein extract subjected to PS (pH 12), respectively.

Since pea proteins were found to be extensively denatured during extraction, as demonstrated by the FTIR (Table 1) and DSC results (Paragraph 3.1.3), it can be concluded that PS and HPH treatments acted on the aggregation state of the denatured extracted proteins. In other words, PS and HPH could promote the exposure of free sulfhydryl groups, hydrophilic and hydrophobic residues on the protein surface following the breakage of aggregates of already denatured proteins stabilized by S-S bridges, hydrogen bonds, electrostatic and hydrophobic interactions. The combined treatment (PS + HPH) led to a lower exposure of free-SH groups as compared to the PS treatment alone, associated with a soluble protein content and H_0_ values comparable to those obtained upon PS, suggesting that the application of HPH after PS favored the formation of novel intermolecular interactions, mainly based on S-S bonds. In this regard, it has been previously observed that intense treatments, such as HPH at 150 MPa for multiple passes applied by Fayaz et al. (2019) on soybean okara or Melchior et al. (2022) on commercial pea protein concentrates, may cause the release and subsequent reaggregation of proteins.

#### 3.1.5 Particle size

**Fig. 2:**
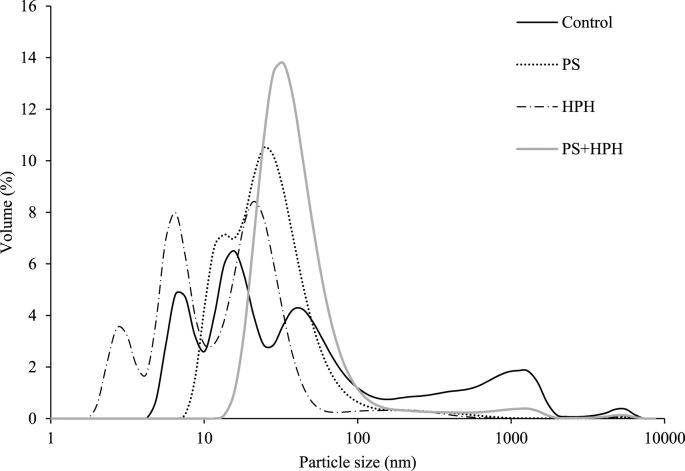
Particle size distribution of pea protein extracts subjected to pH shift (PS), high pressure homogenization (HPH), and their combination (PS + HPH). Data relevant to the untreated pea protein extract (control) are also shown.

To confirm the effect of the treatments on the aggregation state of the pea protein extracts, DLS was conducted (Fig. 2).

The control sample exhibited a very polydisperse distribution characterized by 3 main peaks centred at 4, 15 and 43 nm, and 2 minor peaks at 1280 and 5560 nm, indicating the presence of large aggregates of proteins and/or fibers. PS application resulted in a remarkably higher particle dispersion homogeneity, along with the reduction of both the number of peaks and the associated particle size. Indeed, in this case, most of the particles were comprised under a peak centred at around 20 nm, while the high-dimension peaks observed in the control were substituted by a tail ranging from 100 nm up to 1000 nm, covering a minor particle amount. Such results are in agreement with Jiang et al. (2017) and Yildiz et al. (2017), who highlighted a particle size reduction for pea and soy proteins subjected to PS at pH 12 compared to the untreated sample. These results corroborate the ability of PS treatment to detach aggregate particles. HPH also caused a significant downsizing of the protein extract particles, with three main peak families centred at around 3, 6 and 24 nm, and a further shift of the high-dimension tail at values in the range 50–500 nm. These results agree with the downsizing effect of the intense mechanical stresses, shear forces, and cavitation occurring during HPH of protein extracts from different origins (Fayaz et al., 2019; Luo et al., 2022a; Melchior et al., 2022). Also, HPH has been reported to promote the downsizing of fiber particles (Renoldi et al., 2023). Interestingly, the application of PS + HPH caused the shifting of the particle size peaks towards higher values as compared to those obtained upon single PS or HPH. In particular, the particles in this sample were mostly comprised of a single class centred at about 40 nm, confirming the fact that HPH applied after PS caused the formation of aggregates.

#### 3.1.6 Mechanistic interpretation

**Fig. 3:**
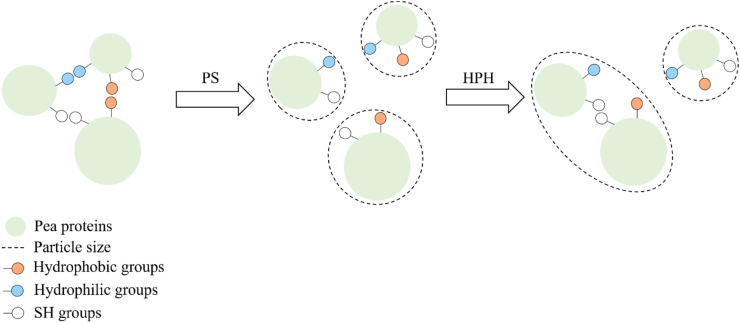
Schematic representation of the effect of pH shift (PS) and high pressure homogenization (HPH) on the structure of pea proteins extracted using alkaline extraction and isoelectric precipitation.

Based on the collected structural information, Fig. 3 reports the hypothesized effect of the treatments of PS and HPH on pea proteins extracted by alkaline extraction and isoelectric precipitation.

The conditions applied during the extraction procedure led to extensive denaturation of native pea proteins, which strongly interacted driven by hydrophilic, hydrophobic and S-S interactions. The application of PS, as well as of HPH to these denatured proteins was not able to alter the primary or the secondary structure of the proteins but rather modified their aggregation state, thus exposing both free SH groups and hydrophobic aromatic residues previously involved in the stabilization of protein aggregates. When the combined treatment was applied, it can be inferred that the small particles formed upon PS further interacted under the intense mechanical stresses generated during HPH, which favored the formation of new S-S bonds.

### 3.2 Effect of PS and HPH on pea protein techno-functional properties

In the second part of the study, the effect of the structural changes induced by the application of the different treatments of PS, HPH and their combination on the techno-functional properties of pea protein extracts was evaluated (Table 3).

**Table 3:** Total solubility, emulsion ability index (EAI), emulsion stability index (ESI), foaming ability index (FAI), foaming stability index (FSI), water holding capacity (WHC), and oil holding capacity (OHC), and antioxidant activity (AA) of pea protein extracts subjected to pH shift (PS), high pressure homogenization (HPH), and their combination (PS + HPH). Data relevant to the untreated pea protein extract (control) are also shown. TE = Trolox equivalents.

Treatment	Total solubility (%)	EAI (m^2^/g)	ESI (min)	FAI (%)	FSI (%)	WHC (g_water_/g_extract_)	OHC (g_oil_/g_extract_)	AA (μg_TE_/g_extract_)
Control	36.6 ± 1.7^c^	2.5 ± 0.1^b^	181.0 ± 23.3^c^	ND	ND	2.62 ± 0.26^c^	5.90 ± 0.33^c^	7.74 ± 0.56^c^
PS	74.7 ± 0.7^a^	4.0 ± 0.3^a^	138.4 ± 6.3^c^	62.8 ± 6.6^b^	73.6 ± 6.0^b^	5.06 ± 0.04^a^	6.52 ± 0.01^b^	9.50 ± 0.49^b^
HPH	53.6 ± 4.2^b^	3.2 ± 0.2^b^	248.1 ± 9.3^b^	29.8 ± 1.7^c^	94.6 ± 0.2^a^	5.24 ± 0.18^a^	7.88 ± 0.23^a^	14.10 ± 0.75^a^
PS + HPH	55.7 ± 1.7^b^	3.0 ± 0.1^b^	547.4 ± 12.5^a^	128.3 ± 7.0^a^	43.1 ± 2.4^c^	4.31 ± 0.02^b^	5.69 ± 0.23^c^	9.57 ± 0.49^b^

^a-d^ means within the same column indicated by different letters are significantly different (p < 0.05). ND = not determined.

#### 3.2.1 Protein solubility

As expected by the soluble protein content of the extracts (Table 2), all the treatments accounted for a significant increase in the overall extract solubility (Table 3), in line with previous studies on plant proteins derived from pumpkin, soybeans, pea and quinoa (Jiang et al., 2018; Luo et al., 2022a, 2022b; Yildiz and Yıldız, 2023).

#### 3.2.2 Interfacial properties

PS treatment significantly enhanced the emulsifying ability of the extracts, while not affecting emulsion stability, in agreement with results reported by Shen et al. (2022) on isolated pea proteins. On the opposite, no significant improvement of EAI was obtained upon HPH applied alone or in combination with PS, while these treatments significantly increased the emulsion stability. These results are consistent with Yildiz et al. (2017), who demonstrated that the combination of HPH (250 MPa) and PS (pH 12) was more effective than individual treatments in stabilizing emulsions prepared with quinoa protein extracts. Regarding foaming properties, the untreated sample exhibited poor foaming capacity, leading to the formation of a thin, non-measurable surface foam layer, in agreement with Liu et al. (2020). PS and HPH treatments applied alone or in combination significantly improved foaming activity, in line with the results of Shen et al. (2022) and Luo et al. (2022a) on pea and quinoa proteins. Overall, these technologies, whether applied individually or in combination, improved the interfacial properties of pea protein extracts, which might be attributed to different mechanisms: (i) reduction of protein and fiber aggregate dimensions (Fig. 2) and increase in protein and fiber solubility (Table 2), which promote rapid diffusion and even distribution at interfaces (Cai et al., 2021; Karim et al., 2008; Luo et al., 2022a; Renoldi et al., 2023; Hua et al., 2017); (ii) exposure of hydrophilic and hydrophobic groups (Table 2), thereby improving the amphiphilic nature of proteins and thus their ability to stably position at the air-oil and air-water interfaces (Manoi and Rizvi, 2009; Pietrysiak et al., 2018).

#### 3.2.3 Water and oil holding capacity

The individual treatments of PS and HPH significantly increased the capacity of the protein extracts to hold both water and oil as compared to the control sample, as shown by the WHC and OHC values, while their combination had a less pronounced effect. The increased WHC suggests that the insoluble fraction was more prone to physically interact with water upon PS and HPH, despite the higher total solubility (Table 3). This could also explain the increase in OHC, which would be further supported by both the lower particle size (Fig. 2) and the higher hydrophobicity (Table 2) (Yildiz et al., 2017; Yildiz & Yildiz, 2023).

#### 3.2.4 Antioxidant activity

The PS and HPH treatments, also in combination, increased the antioxidant activity of the protein extracts (Table 3), as already shown in the literature (D'Alessio et al., 2023; Pirozzi and Donsì, 2023). This can be attributed to the ability of the treatments to increase the exposure of free SH groups (Table 2), which can act as proton donors to electron-lacking radicals (Žilić et al., 2012).

#### 3.2.5 PCA

**Fig. 4:**
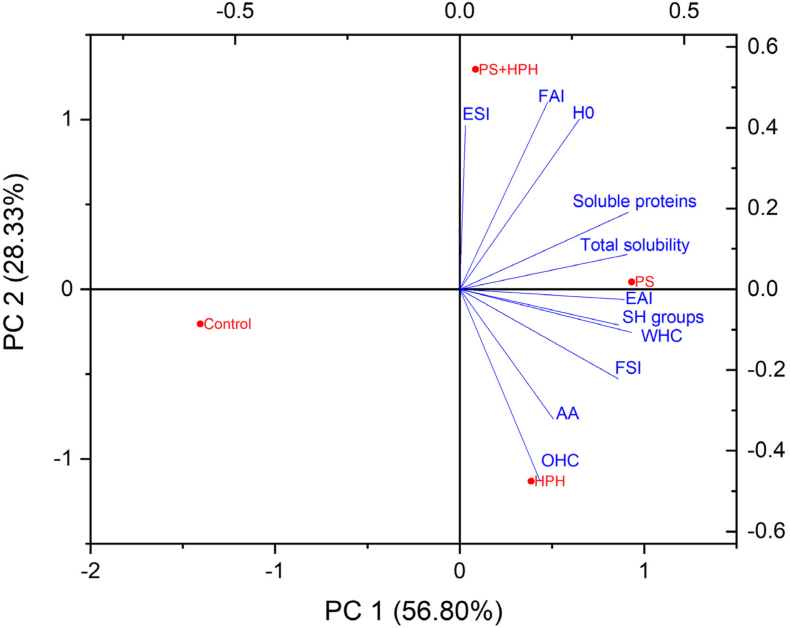
PCA biplot of structural and techno-functional properties of pea protein extracts subjected to pH shift (PS), high pressure homogenization (HPH), and their combination (PS + HPH). Data relevant to the untreated pea protein extract (control) are also shown. H_0_ = surface hydrophobicity, EAI = emulsion activity index, ESI = emulsion stability index, FAI = foaming activity index, FSI = foaming stability index, WHC = water holding capacity, OHC = oil holding capacity, AA = antioxidant activity.

Principal component analysis (PCA) was performed to have an overview of the effect of structural changes induced by PS, HPH and their combination on the structural and technological properties of the protein extracts. In particular, PCA analysis took into account the amount of free SH groups and surface hydrophobicity (Table 2), and the extract techno-functional properties (Table 3). Fig. 4 reports the biplot obtained from the first two principal components, which respectively explained 56 % and 28 % of the total variance, for a total contribution of 85.1 %.

The control protein extract was positioned in the left negative quadrant, opposite to those subjected to PS, HPH or PS + HPH, indicating that these treatments had a significant effect on both the structural and the techno-functional properties of pea proteins. All the considered variables tended unambiguously towards the treated extracts, indicating an increase upon the applied technological interventions. The HPH-treated sample was particularly effective in improving OHC and AA, while PS promoted the increase of total solubility, soluble protein content, EAI, WHC, and SH groups. The PS + HPH treatment was positioned in the upper positive quadrant, confirming that the combined intervention had an effect different from that of the single ones, resulting in the improvement, especially, of ESI, FAI, and H_0_.

## 4 Conclusions

In this study, technological interventions of pH-shift and high-pressure homogenization were applied to proteins extracted from peas discarded at the industrial level to obtain ingredients with tailored functionalities. The analyses conducted on the pea protein extracts allowed concluding that, despite the proteins being extensively denatured during extraction, all the technological interventions acted on protein aggregation, releasing smaller protein particles and exposing hydrophilic and hydrophobic residues, as well as free SH groups, previously involved in aggregate stabilization. Such disaggregation significantly improved protein extract solubility, interfacial properties, and water and oil holding capacity and also led to an increase in their antioxidant activity. Besides the pivotal role of protein, it could not be excluded the contribution of other minor components, mainly fibers, in determining the extract techno-functional properties.

Overall, the obtained results demonstrate the possibility of up-cycling by-products of the legume industry to produce protein-rich ingredients with improved functional properties by the application of technological interventions of pH-shift and high-pressure homogenization and their combination. Remarkably, each intervention resulted in tailored improvement of protein solubility, interfacial properties or ability to hold water and oil, which could be exploited to design a specific process based on the required techno-functional properties.

It should also be noted that the proposed approach to protein functionalization does not compromise extraction yield, as the treatments have been applied post-extraction, showing the ability to effectively modulate techno-functional properties even when the proteins are already denatured. In the future, gentler extraction and purification methods could be tested to better preserve protein native structure, possibly further enhancing the effect of the proposed technologies aiming at obtaining plant protein ingredients with high functionalities.

## CRediT author statement

**Stella Plazzotta**: Conceptualization, funding acquisition, project administration, resources, supervision, writing-review and editing; **Sofia Melchior**: data curation, formal analysis, investigation, methodology, visualization, writing-original draft, writing-review and editing; **Lorenzo Barozzi**: data curation, formal analysis, writing-review and editing; **Alberto Saitta**: writing-original draft, writing-review and editing; **Maria Cristina Nicoli**: resources, writing-review and editing; **Lara Manzocco**: resources, supervision, writing-review and editing.

## Funding

This work was financed by the EU- NextGenerationEU Project “Upcycling pea waste side streams for developing future food ingredients -UPea”; PRIN Bando 2022; Prot. 20222P5C3E.

**Figure:**
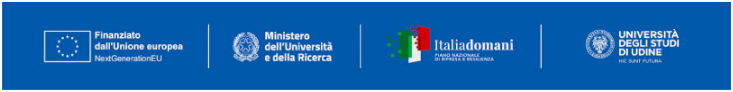

## Declaration of competing interest

The authors declare that they have no known competing financial interests or personal relationships that could have appeared to influence the work reported in this paper.

## Data Availability

Data will be made available on request.
