# Supplementary material for: Effect of pH shift and high pressure homogenization on the structure and techno-functional properties of protein extracts from substandard peas
Source: Curr Res Food Sci. 2025 Dec 20;12:101286. doi: 10.1016/j.crfs.2025.101286 (PMC12810341; doi:10.1016/j.crfs.2025.101286)
Supplement: Multimedia component 1 [file mmc1.docx]

Table S1. Protein and fiber content of pea protein extracts subjected to pH shift (PS), high pressure homogenization (HPH), and their combination (PS+HPH). Data relevant to the untreated pea protein extract (control) are also shown.

| Treatment | Total proteins (g/100 g_extract_) | Total fiber (g/100 g_extract_) |
| --- | --- | --- |
| Control | 70.8 ± 0.6^a^ | 8.5 ± 1.8^a^ |
| PS | 68.9 ± 0.5^a^ | 9.6 ± 0.7^a^ |
| HPH | 69.9 ± 0.1^a^ | 12.2 ± 2.3^a^ |
| PS+HPH | 70.1 ± 0.3^a^ | 11.1 ± 1.7^a^ |

^a-d^ means within the same column indicated by different letters are significantly different (p < 0.05).


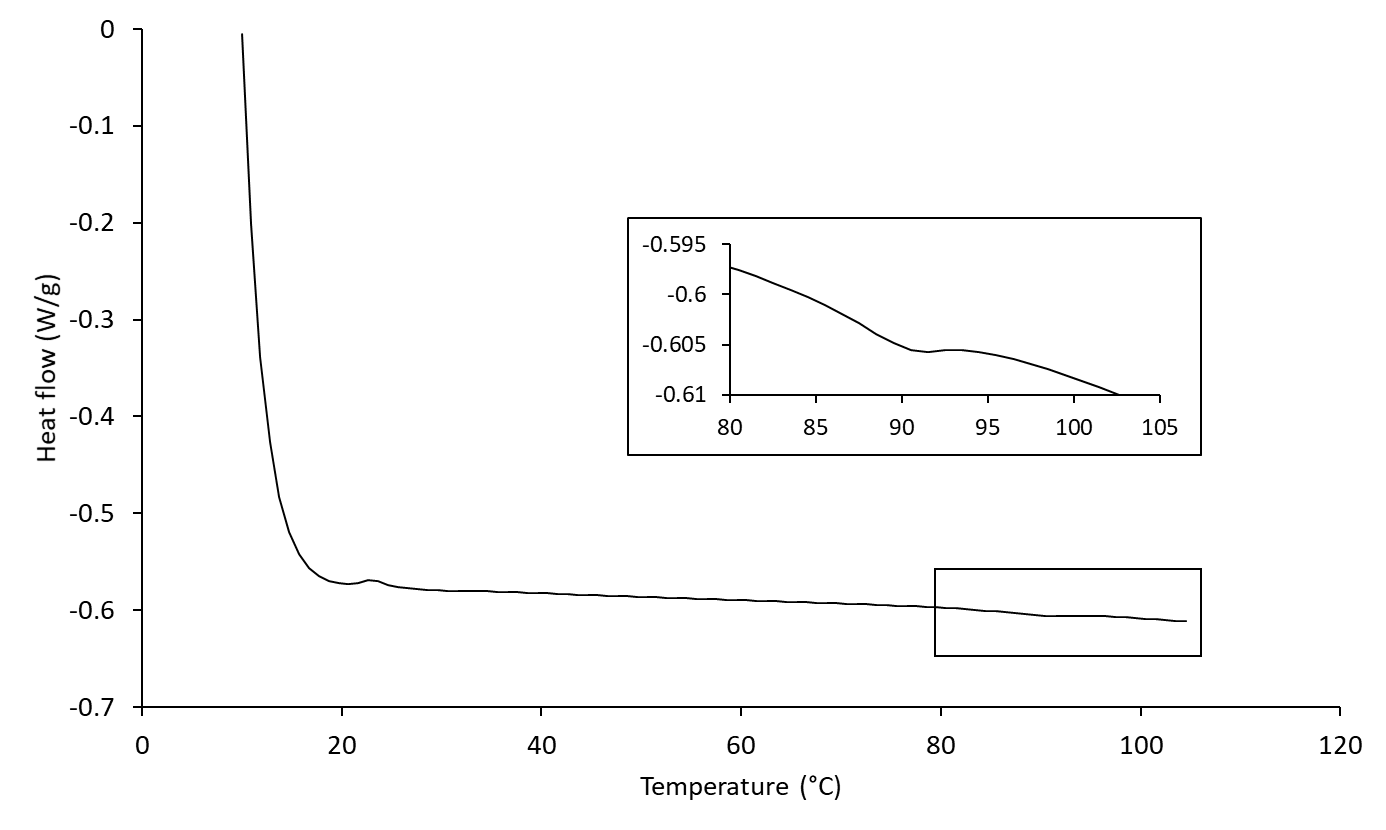


Figure S1. Thermograms obtained by DSC analysis of untreated pea protein extracts (control). The inset shows the denaturation peak. Thermograms relevant to extracts subjected to pH shift (PS), high pressure homogenization (HPH), and their combination (PS+HPH) are not shown since flat.
